# Differential TLR activation of murine mesenchymal stem cells generates distinct immunomodulatory effects in EAE

**DOI:** 10.1186/s13287-016-0402-4

**Published:** 2016-10-10

**Authors:** Ana María Vega-Letter, Mónica Kurte, Catalina Fernández-O’Ryan, Melanie Gauthier-Abeliuk, Patricia Fuenzalida, Ivón Moya-Uribe, Claudia Altamirano, Fernando Figueroa, Carlos Irarrázabal, Flavio Carrión

**Affiliations:** 1 Laboratory of Cellular and Molecular Immunology, Faculty of Medicine, Universidad de los Andes, Monseñor Álvaro del Portillo N°12.455, Las Condes, Santiago, 750000 Chile; 2 Cell Culture Laboratory Animals, School of of Biochemical Engineering, Pontificia Universidad Católica de Valparaíso, Av. Brasil 2085, Valparaíso, 2362803 Chile; 3 Laboratory of Integrative and Molecular Physiology, Faculty of Medicine, Universidad de los Andes, Monseñor Álvaro del Portillo N°12.455, Las Condes, Santiago, 750000 Chile

**Keywords:** Mesenchymal stem cells, Toll-like receptors 3 and 4, Experimental autoimmune encephalomyelitis, Autoimmunity, Immunomodulation

## Abstract

**Background:**

Recently, it has been observed that mesenchymal stem cells (MSCs) can modulate their immunoregulatory properties depending on the specific in-vitro activation of different Toll-like receptors (TLR), such as TLR3 and TLR4. In the present study, we evaluated the effect of polyinosinic:polycytidylic acid (poly(I:C)) and lipopolysaccharide (LPS) pretreatment on the immunological capacity of MSCs in vitro and in vivo.

**Methods:**

C57BL/6 bone marrow-derived MSCs were pretreated with poly(I:C) and LPS for 1 hour and their immunomodulatory capacity was evaluated. T-cell proliferation and their effect on Th1, Th17, and Treg differentiation/activation were measured. Next, we evaluated the therapeutic effect of MSCs in an experimental autoimmune encephalomyelitis (EAE) model, which was induced for 27 days with MOG_35–55_ peptide following the standard protocol. Mice were subjected to a single intraperitoneal injection (2 × 10^6^ MSCs/100 μl) on day 4. Clinical score and body weight were monitored daily by blinded analysis. At day 27, mice were euthanized and draining lymph nodes were extracted for Th1, Th17, and Treg detection by flow cytometry.

**Results:**

Pretreatment of MSCs with poly(I:C) significantly reduced the proliferation of CD3^+^ T cells as well as nitric oxide secretion, an important immunosuppressive factor. Furthermore, MSCs treated with poly(I:C) reduced the differentiation/activation of proinflammatory lymphocytes, Th1 and Th17. In contrast, MSCs pretreated with LPS increased CD3^+^ T-cell proliferation, and induced Th1 and Th17 cells, as well as the levels of proinflammatory cytokine IL-6. Finally, we observed that intraperitoneal administration of MSCs pretreated with poly(I:C) significantly reduced the severity of EAE as well as the percentages of Th1 and Th17 proinflammatory subsets, while the pretreatment of MSCs with LPS completely reversed the therapeutic immunosuppressive effect of MSCs.

**Conclusions:**

Taken together, these data show that pretreatment of MSCs with poly(I:C) improved their immunosuppressive abilities. This may provide an opportunity to better define strategies for cell-based therapies to autoimmune diseases.

## Background

Mesenchymal stem cells (MSCs) are nonhematopoietic, multipotent progenitor cells isolated from a variety of adult tissues, including bone marrow and adipose tissue. They are capable of self-renewal and are able to differentiate into at least some mesenchymal cell types, such as bone, cartilage, and fat, thus playing a potential role in tissue repair [1, 2]. In addition to their potential for differentiation, MSCs also exhibit immunosuppressive activity, as shown by their ability to inhibit the proliferation and function of immunocompetent cells, such as T and B lymphocytes, natural killer cells, and dendritic cells [3–5]. These immunomodulatory properties of MSCs have generated great interest in their potential as a promising therapeutic modality for proinflammatory and autoimmune diseases [6, 7]. Diverse studies using experimental animal models have shown that MSCs can reduce the progression and/or severity of various immune-mediated diseases, such as collagen-induced arthritis (CIA) [8], experimental colitis [9], and experimental autoimmune encephalomyelitis (EAE) [10, 11]. In addition, we recently demonstrated that the intravenous administration of MSCs in EAE at different stages of the disease induced differential therapeutic effects depending on the proinflammatory environment at each stage of the disease [12].

It has also been demonstrated that the immunosuppressive activity of MSCs does not seem to be spontaneous but instead requires MSCs to be “licensed” in an appropriate proinflammatory environment to exert their effects [13, 14]. In this line, Krampera et al. and Ren et al. showed that MSCs mediating immunosuppression required preliminary activation by immune cells through the secretion of the proinflammatory cytokine IFN-γ, either alone or together with TNF-α, IL-1α, or IL-1β [3, 15]. These cytokine combinations induced the MSCs to express high levels of soluble factors involved in MSC-mediated immunosuppression, such as indoleamine 2,3-dioxygenase (IDO), transforming growth factor beta (TGF-β), prostaglandins, and nitric oxide (NO), as well as other factors [3, 15–17].

In addition to activation of MSCs by proinflammatory cytokines, Toll-like receptors (TLRs) can influence their immunomodulatory capacity. For example, Liota et al. [18] showed that human bone marrow-derived MSCs (BM-MSCs) express high levels of TLR3 and TLR4, and that ligation of these receptors by their agonists, polyinosinic:polycytidylic acid (poly(I:C)) and lipopolysaccharide (LPS), respectively, can reduce the inhibitory activity of MSCs on CD4^+^ T-cell proliferation. In contrast, Opitz et al. showed that pretreatment of human BM-MSCs for 24 hours with poly(I:C) or LPS significantly enhanced the immunosuppressive activity of BM-MSCs on the allo-mixed lymphocyte reaction (MLR) [19]. On the contrary, recent results demonstrate that human MSCs polarize into two active phenotypes following specific TLR3 or TLR4 activation. Priming by TLR3 agonists specifically leads to the expression of immune dampening mediators and the maintained suppression of T-cell activation. In contrast, priming by TLR4 agonists results in the expression of proinflammatory mediators and a reversal of the MSC-established suppressive mechanisms of T-cell activation [20]. Besides these in-vitro studies, our group recently demonstrated that TLR3 preconditioning increases the therapeutic efficacy of human umbilical cord MSCs in a mouse model of colitis [21]. These results demonstrate the complexity of the immunomodulatory capacity of MSCs and suggest that TLR activation may affect the functional immune activity of MSCs.

The aim of the present study was to demonstrate, for the first time in murine MSCs and an experimental model of multiple sclerosis (EAE), that in-vitro pretreatment of MSCs with poly(I:C) or LPS can induce two distinct active phenotypes in MSCs, as found in humans, and that these polarized cells possess opposite immunological effects in vitro and in vivo. Our results indicate that pretreatment of MSCs with poly(I:C) enhances their immunosuppressive capacity on T lymphocytes and that the intraperitoneal injection of these MSCs significantly reduces the severity of EAE. In contrast, LPS-pretreated MSCs induced a significant increase in T-cell proliferation and completely reversed the immunosuppressive therapeutic effect of MSCs in EAE.

## Methods

### Animals

Female C57BL/6 mice, 8–14 weeks old, were purchased from the central animal facility of the Faculty of Medicine, University of Chile. Animals were housed under standard laboratory conditions and provided food and water ad libitum. Experimental procedures and protocols were performed according to the US National Institute of Health Guide for the care and use of laboratory animals (NIH Publication No. 85-23, revised 1996), and were approved by the institutional animal care and use committee of the Universidad de los Andes and the FONDECYT bioethics advisory committee in Chile.

### MSC culture in vitro

MSCs (GIBCO Mouse (C57BL/6), catalog number S1502-100) were obtained from bone marrow isolated from C57BL/6 mice at ≤ 8 weeks of age through mechanical and enzymatic digestion. Cells were cultured in complete alpha modified Eagle’s medium (αMEM; Gibco, Auckland, New Zealand) containing 10 % heat-inactivated MSCs, qualified fetal bovine serum (FBS; Gibco), 100 U/ml penicillin, and 100 μg/ml streptomycin (Gibco), and incubated at 37 °C with 5 % CO_2_. At subconfluence, cells were replated at a density of 5000 cells/cm^2^ and used between passages 9 and 12.

### MSC characterization

The MSC phenotype was confirmed by flow cytometry based on the positivity for CD29, CD44, and Sca-1, in the absence of CD45 and C11b antigen. All antibodies were purchased from BD Biosciences (San Diego, CA, USA). Surface staining was performed following a standard protocol. The samples were acquired with a FACSCanto II flow cytometer (Becton, Dickinson and Company). Data were analyzed using FCS Express 4 Plus research edition and Flow Jo software. Determination of the capacity of MSCs to differentiate toward chondrogenic, adipogenic, and osteogenic lineages was performed as described previously [22].

### Treatment of MSCs

MSCs were grown to 70–80 % confluence and incubated with agonists for 1 hour in complete αMEM. For TLR pretreatment of MSCs, poly(I:C) (10 μg/ml; Sigma-Aldrich, Israel) and LPS (500 ng/ml; Sigma-Aldrich, Israel) were used as agonists for TLR3 and TLR4, respectively. Cells were then washed thoroughly with a complete cell culture medium before use in the different assays described in the following.

### Quantitative real-time PCR analysis

For the evaluation of MSC gene expression, after pretreatment with poly(I:C) and LPS for 1 hour, cells were thoroughly washed and cultured for 12 hours in complete αMEM. Cells were then harvested using Trypsin 1× (Trypsin–EDTA 1×; Gibco) and pelleted. Total RNA was isolated using the RNeasy Mini Kit (Qiagen) following the manufacturer’s instructions. The RNA concentration was measured with a NanoDrop 2000 spectrophotometer (Thermo Scientific), and cDNA was synthesized from 2 μg of RNA using a reverse transcription protocol (Improm II-RT, A3802; Promega). Quantitative real-time PCR (RT-qPCR) was performed in a Stratagene MX3000P thermocycler (Agilent Technologies) using GoTaq qPCR Mastermix (Promega, Madison, WI, USA). 18S was used to normalize the results, and basal conditions were used for calibration. MxPro v4.10d software was used for analysis using the 2^–(ΔΔCt)^ formula, where ΔΔCt takes into account the efficiency of the primers and the normalized Ct values. The primer sequences used for amplification are presented in Table 1.

**Table 1:** mRNA primer information

Gene name	Forward primer (5ʹ a 3′)	Reverse primer (5′ a 3′)	Access Number Gene Bank
TLR3	AAAACTCAGCGGCCAGGAAT	AGTTACGAAGAGGGCGGAAA	NM_126166.4
TLR4	TGGCTGGTTTACACGTCCAT	GCAGAAACATTCGCCAAGCA	NM_021297.2
18s	ATCGCCAGTCGGCATCGTTTAT	GCCGCTAGAGGTGAAATTCTTGGA	NR_003286.2

*Abbreviations*: *TLR3*, toll like receptor 3; *TLR4*, toll like receptor 4; *18S*, 18S ribosomal RNA

### Proliferation assays

Splenocytes were obtained from the spleen of adult C57BL/6 mice. Extracted cells were passed through a 70-μm filter (cell strainer; BD Falcon), centrifuged at 1680 rpm for 6 minutes, and treated with cold NH_4_Cl for 5 minutes. Cells were then washed in PBS (phosphate-buffered saline) and centrifuged at 1680 rpm for 6 minutes. Next, cells were labeled with CellTrace Violet (CTV) (Invitrogen, UK) according to the manufacturer’s instructions. For T-cell activation, 2 × 10^5^ cells were stimulated with concanavalin A (conA) (0.5 μg/ml) in the presence or absence of MSCs at a 1:10 ratio (MSCs:splenocytes) in complete RPMI medium with 10 % FBS, 100 U/ml penicillin, and 100 μg/ml streptomycin (Gibco) at 37 °C in a 5 % CO_2_ atmosphere. After 5 days of culture, cells were washed and evaluated by flow cytometry for the percentage of CD3^+^ T cells in the population. For the proliferation analysis, we used CTV, which functions similarly to standard CFSE staining. CTV was added at the beginning of the cultures. Each peak on the histograms corresponds to the division cycles for CD3^+^ lymphocytes. After obtaining the number of events, we calculated a proliferation index that incorporated the number of cells divided by the number of progenitors as described by Roederer [23].

### In-vitro T-helper cell differentiation

Purified CD4^+^ T cells were isolated from C57BL/6 splenocytes using the Dynabeads untouched mouse CD4 Cell kit (Invitrogen) according to the manufacturer’s instructions. Purified CD4^+^ T lymphocytes were cultured in RPMI Medium 1640 with GlutaMAX supplemented with 10 % FBS, 1 units/ml penicillin/0.1 mg/ml streptomycin, 1 mM sodium pyruvate, 20 mM HEPES, and 50 μM of β-mercaptoethanol (Invitrogen, Grand Island, NY, USA). T cells were stimulated in 48-well plates coated with anti-CD3 (2 μg/ml) and anti-CD28 (1 μg/ml) antibodies (BD Biosciences, San Diego, CA, USA) and were subjected to specific T-cell differentiation. Th1 differentiation was induced by adding 5 ng/ml of IL-12 and 2.5 μg/ml of anti-IL-4. Th17 differentiation was induced by adding TGF-β (5 ng/ml), IL-6 (50 ng/ml), and IL-23 (5 ng/ml) and neutralizing antibodies for IFN-γ and IL-4 (2.5 μg/ml each). Each stimulation period lasted 5 days. T cells were plated into the wells, and MSCs were added in a 1:10 ratio (MSCs:CD4^+^ T cells). For FACS analysis, differentiated Th1 and Th17 cells were restimulated with PMA/ionomycin for 3.5 hours in the presence of brefeldin for the last 2.5 hours of incubation at 37 °C before antibody staining.

### Th1 and Th17 FACS analysis

Differentiated T cells were stained with an anti-CD4 PE-conjugated antibody (BD Biosciences) for 30 minutes at 4 °C in staining buffer. Intracellular staining was performed using a CytoFix/Cytoperm kit (BD Bioscience) following the manufacturer’s instructions. Cells were stained with anti-IFN-γ (FITC-conjugated) antibody for the Th1 subset of the population, or an anti-IL-17A (PE-conjugated) antibody for the Th17 subset of the population. After membrane and intracellular staining, cells were analyzed with a FACSCanto II using the FACS Express software. For the proliferation analysis, we used CTV, which functions similarly to standard CFSE staining. CTV was added at the beginning of the cultures. Each peak on the histograms corresponds to the division cycles for CD4^+^IFN-γ^+^ and CD4^+^IL-17^+^ lymphocytes, corresponding to Th17 and Th1 lymphocytes, respectively. After obtaining the number of events, we calculated a proliferation index that incorporated the number of cells divided by the number of progenitors.

### EAE induction and treatment

Female C57BL/6 mice (10–14 weeks old) were injected subcutaneously (s.c.) in the flank with 50 μg of MOG_35–55_ peptide (LifeTein LCC, USA) emulsified in a complete Freund’s adjuvant (Difco Laboratories, Detroit, MI, USA) supplemented with heat-inactivated *Mycobacterium tuberculosis* H37RA (Difco Laboratories). Subsequently, 2 and 48 hours later, mice received 350 ng of pertussis toxin (Calbiochem, La Jolla, CA, USA) intraperitoneally (i.p.). Clinical signs appeared 10 days after EAE induction as described previously [24] . Thus, to evaluate the therapeutic effect of untreated MSCs or MSCs pretreated with poly(I:C) and LPS, mice were injected i.p. on day 4 with 2 × 10^6^ MSCs in 100 μl of PBS.

### Score analysis

Mice were monitored daily by a blinded observer for behavioral EAE symptoms, scored, and weighed, as reported previously [12], for 27 days. Classical EAE scores were assigned as follows: 0 = no disease; 0.5 = reduced tail tonus; 1 = limp tail; 1.5 = limp tail and ataxia; 2 = limp tail, ataxia, and hind-limb weakness; 2.5 = at least one hind limb paralyzed/weak; 3 = both hind limbs paralyzed/weak; 3.5 = complete paralysis of hind limbs; 4 = paralysis to hip; and 5 = moribund or dead.

### ELISA for cytokines

Culture supernatants were assayed for IL-6 using an ELISA kit (catalog number DY406; R&D systems) according to the manufacturer’s protocol.

### Measurement of iNOS activity

NO was detected using a modified Griess reagent (Sigma-Aldrich). Briefly, all NO_3_ was converted into NO_2_ by nitrate reductase, and total NO_2_ was detected by the Griess reaction as described previously [25].

### Ex-vivo T-cell analysis

For ex-vivo T-cell analyses, draining inguinal and axillary lymph nodes were removed from mice 27 days after EAE induction. T cells were obtained and cultured at a density of 2.5 × 10^5^/well. Inflammatory cells were restimulated with PMA/ionomycin for 3.5 hours in the presence of brefeldin A for the last 2.5 hours of incubation at 37 °C before antibody staining and analysis by flow cytometry. Next, Th1 and Th17 cells in the samples from the different groups were identified as already described. Finally, after membrane and intracellular staining, cells were analyzed with a FACSCanto II using the FACS Express software.

### Statistical analysis

A Kruskal–Wallis test, which accounts for non-normal distributions with small sample sizes and multiple groups, was performed for comparisons between experimental groups. Post-hoc analyses were performed with the Mann–Whitney test. For all analyses, we used GraphPad Prism Program (GraphPad, San Diego, CA, USA) statistical software. *p* < 0.05 was considered statistically significant. Data are presented as the mean ± standard deviation.

## Results

### Characterization and TLR expression of MSCs

**Fig. 1:**
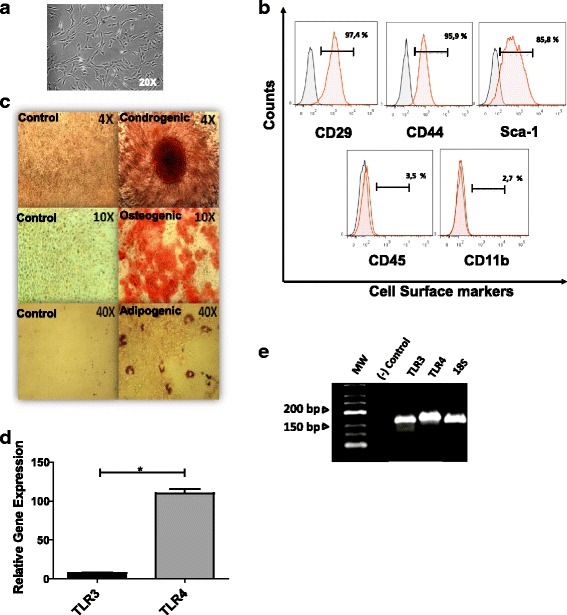
Characterization of murine MSCs and TLR expression. (**a**) Morphology and characteristics of murine BM-MSCs (20× magnification). (**b**) Immunophenotypic profile from a representative murine MSC population. Cell surface markers, *solid red*; isotype cell controls, *gray*. (**c**) MSCs were confirmed to have the capacity to differentiate into chondrocytes, osteoblasts, and adipocytes (*right*) by alizarin red, safranin O, and oil red staining (4, 10, and 40× magnification, respectively), as described in Methods. Respective controls (culture medium only, without differentiation conditions) (*Left*). (**d**) Relative expression of TLR3 and TLR4 in MSCs confirmed by RT-qPCR normalized with 18S = 1 × 10^–5^ as described previously [37] (*n* = 3). (**e**) RT-qPCR products were analyzed by 2.5 % agarose gel electrophoresis along with a low-range DNA ladder (MW). Negative control, without cDNA template. *MW* molecular weight. *p < 0.05, **p < 0.01, ***p < 0.001

Murine MSCs were cultured in complete αMEM for the selective proliferation of MSCs. After culturing, cells with a stable fibroblast-like phenotype were used for experimentation (Fig. 1a). As evidenced by flow cytometry, cells were uniformly and strongly positive for MSC-related markers, such as CD44, CD29, and Sca-1 (80–99 %), and were negative for CD45 and CD11b (<4 %) (Fig. 1c). As shown in Fig. 1c, we confirmed the ability of MSCs to differentiate into adipocytes, chondrocytes, and osteoblasts using a specific differentiation stimulus (right) or control medium (left) as described in Methods. We next examined the relative expression of TLR3 and TLR4 genes in MSCs using RT-qPCR and gel electrophoresis. RT-qPCR and agarose gel electrophoresis analysis revealed that murine MSCs expressed both TLRs and that the expression level of TLR4 was higher than TLR3 (Fig. 1d, e). We also found that pretreatment of MSCs with poly(I:C) and LPS for 1 hour did not affect the immunophenotypic profile of murine MSCs (data not shown).

### TLR3 and TLR4 pretreatment differentially affect the in-vitro immunosuppressive capacity of murine MSCs

To evaluate the effect of specific stimulation of TLR3 and TLR4, we treated the MSCs for 1 hour with poly(I:C) or LPS in complete αMEM and then determined their in-vitro immunomodulatory capacity. First, we tested the immunosuppressive capacity of MSCs to inhibit T-cell proliferation induced by conA. Briefly, splenocytes were isolated from C57BL/6 mice, stained with CTV (a fluorescent dye used to determine T-lymphocyte proliferation), and then stimulated with ConA for 3 days. Flow cytometry was used to analyze the proliferation of CD3^+^ T lymphocytes that were cultured in the presence or absence of untreated MSCs or MSCs pretreated with poly(I:C) or LPS at different MSC:splenocyte ratios (1:5, 1:10, and 1:20), as described in Methods.

**Fig. 2:**
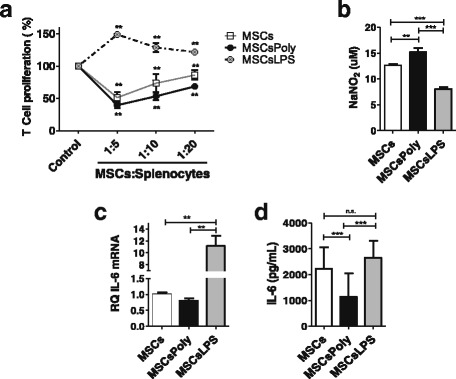
Immunomodulatory capacity of MSCs stimulated with TLR3 and TLR4 ligands in vitro. (**a**) To study the immunosuppressive capacity of untreated MSCs and MSCs pretreated for 1 hour with poly(I:C) or LPS on T-cell response, we performed an in-vitro T-cell stimulation assay at different ratios of MSCs:splenocytes, as described in Methods. MSCs were either unstimulated or were stimulated with poly(I:C) (10 μg/ml) or LPS (500 ng/ml) for 1 hour before being cocultured with T cells in complete RPMI medium. Previously, splenocytes were labeled with CTV and stimulated with Con A and finally cultured with MSCs at 1:5, 1:10, or 1:20 ratios for 3 days. T-cell proliferation was evaluated by flow cytometry, gating on CD3^+^ cells. (**b**) Secretion of nitric oxide (*NO*) by MSCs in coculture with splenocytes at a 1:10 ratio was measured using a modified Griess reagent. (**c**) IL-6 mRNA expression evaluated by RT-qPCR. (**d**) IL-6 secretion, measured by ELISA. Data expressed as the mean ± SED. A Mann–Whitney test was performed, *p < 0.05, **p < 0.01,***p < 0.001. *MSCsPoly* MSCs pretreated with poly(I:C) for 1 hour, *MSCsLPS* MSCs pretreated with LPS for 1 hour

The addition of MSCs to the T-cell cultures significantly decreased the proliferation of CD3^+^ T lymphocytes in a dose-dependent manner compared with proliferation in the control condition (without MSCs) (*p* < 0.05, Fig. 2a). MSCs pretreated with poly(I:C) for 1 hour were considerably more effective than untreated MSCs in inhibiting T-cell proliferation at the different ratios analyzed (*p* < 0.001, Fig. 2a). In contrast, MSCs pretreated with LPS for 1 hour not only reversed the immunosuppressive effect of MSCs but also induced a significant increase in T-cell proliferation in a dose-dependent manner compared with the effects in the control condition (*p* < 0.001, Fig. 2a).

We next studied the effect of poly(I:C) and LPS pretreatment of murine MSCs on the expression of immune modulators, such as the soluble immunosuppressive factors NO and proinflammatory cytokine IL-6. Supernatants derived from MSCs cultured in complete αMEM for hours in the absence or presence of splenocytes were used to evaluate the presence of NO, as described in Methods. A modified Griess assay for nitrite quantitation showed no significant differences in NO secreted by untreated or pretreated MSCs (data not shown). However, when MSCs were cultured in the presence of splenocytes, we observed a significant increase in NO production induced by the MSCs pretreated with poly(I:C) compared with untreated MSCs (*p* < 0.001, Fig. 2b). Conversely, pretreatment of MSCs with LPS induced lower NO production in comparison with untreated MSCs or MSCs pretreated with poly(I:C) (*p* < 0.001, Fig. 2b).

Quantitative analysis of IL-6 expression, evaluated by RT-qPCR, revealed that MSCs pretreated with LPS induced a significant increase in the relative expression of IL-6 compared with untreated MSCs or poly(I:C) pretreated MSCs (*p* < 0.05, Fig. 2c). No significant differences were observed in mRNA IL-6 expression between untreated MSCs and MSCs pretreated with poly(I:C). We also observed that MSCs pretreated with LPS had higher IL-8 mRNA expression than untreated MSCs or MSCs pretreated with poly(I:C) (data not shown). Moreover, we observed that MSCs pretreated with poly(I:C) lose the capacity to secrete IL-6, as measured by ELISA after 24 hours of stimulation, compared with the observed IL-6 secretion in untreated MSCs and MSCs pretreated with LPS (*p* < 0.001, Fig. 2d). These results suggest that MSCs pretreated for 1 h with poly(I:C) have a higher immunosuppressive effect in vitro when compared with untreated MSCs or MSCs pretreated with LPS.

### Pretreatment of murine MSCs with poly(I:C) or LPS induces different and opposing in-vitro effects on Th1 and Th17 subsets

We next studied the immunomodulatory effect of the addition of untreated murine MSCs or MSCs pretreated with poly(I:C) or LPS for 1 hour on in-vitro differentiation and proliferation of Th1 and Th17 lymphocytes. CD4^+^ T lymphocytes, purified by negative selection from splenocytes, were stained with CTV and cultured under Th1 and Th17 polarizing conditions in the absence or presence of TLR3 or TLR4-stimulated MSCs. MSCs were added at the beginning of the differentiation protocol at a 1:10 ratio (MSCs:CD4^+^ T cells) and intracellular cytokines for Th1 and Th17 cells were evaluated at day 5 by flow cytometry, as described in Methods.

**Fig. 3:**
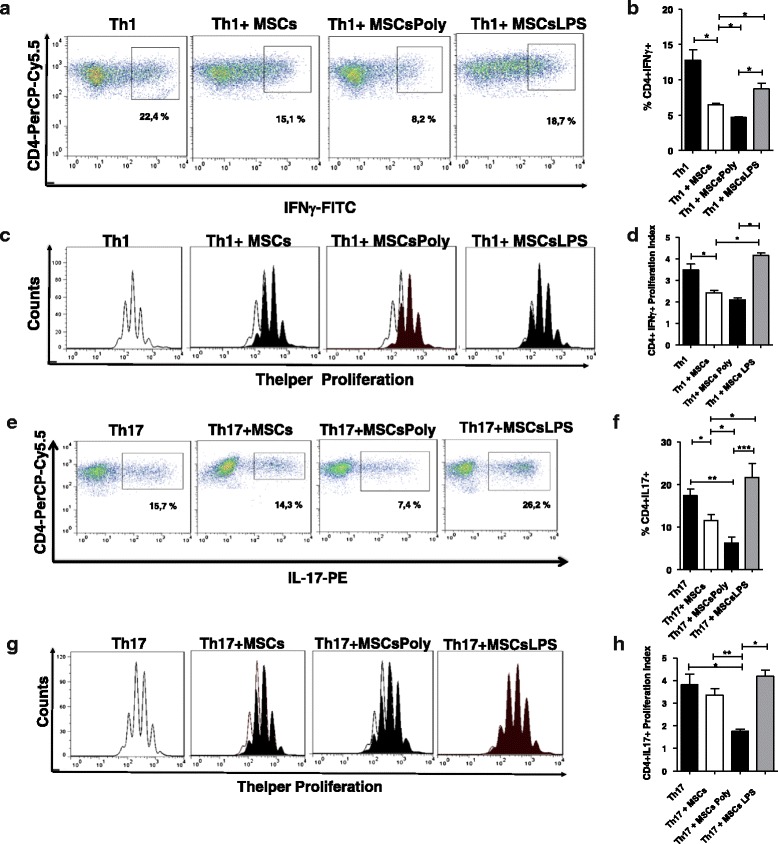
MSCs stimulated with TLR3 and TLR4 ligands differentially modulate Th1 and Th17 differentiation and proliferation. T-helper cell differentiation (**a**, **b**, **e**, **f**) and proliferation (**c**, **d**, **g**, **h**) were assessed using naïve CD4^+^ T cells. Purified CD4^+^ cells were stimulated with a specific cocktail of cytokines, as described in Methods, to induce Th1 (**a**–**d**) and Th17 (**e**–**h**) differentiation in the absence or presence of MSCs pretreated with or without a TLR agonist. MSCs were either unstimulated or were stimulated with poly(I:C) (10 μg/ml) or LPS (500 ng/ml) for 1 hour before being cocultured with CD4^+^ T cells in complete RPMI medium. MSCs were added at day 0 of the differentiation process in a 1:10 ratio (MSCs:T cells). Flow cytometry analysis, gating on CD4^+^ cells, and intracellular staining, using antibodies (*mAb*) for IFN-γ and IL17 to identify Th1 and Th17 lymphocytes, respectively, were performed. Representative density plots of six different experiments for Th1 and Th17 differentiation are shown. For proliferation analysis, CD4^+^ cells were previously labeled with CellTrace Violet (*CTV*) and analyzed (presented as histograms). Further analysis of the events of each cycle, described by the proliferation index (**d**, **h**). Th1 differentiation (**b**) and proliferation (**d**) with the MSCs pretreated with poly(I:C) and LPS. Th17 differentiation (**f**) and proliferation (**h**) with the MSCs pretreated with poly(I:C) and LPS. *Bars* represent the mean ± SEM, significant differences calculated using the Mann–Whitney test. *p < 0.05, **p < 0.001. *MSCsPoly* MSCs pretreated with poly(I:C) for 1 hour, *MSCsLPS* MSCs pretreated with LPS for 1 hour

The patterns of Th1 and Th17 differentiation and proliferation for six different experiments are shown in Fig. 3a, e. The data analysis summary of the proliferation is shown in Fig. 3b, f.

As shown in Fig. 3a, b, the addition of untreated MSCs significantly suppressed the clonal expansion of IFN-γ-secreting (Th1) cells relative to that we reported previously [22]. (*p* < 0.05, Fig. 3a, b). The pretreatment of MSCs with poly(I:C) induced a higher capacity to inhibit Th1 than was observed in untreated MSCs (*p* < 0.05). Although not significantly different, the addition of MSCs treated with poly(I:C) also induced a decrease in the proliferation of Th1 cells compared with that in untreated MSCs (Fig. 3c, d). In contrast, culturing Th1 cells with MSCs pretreated with LPS showed that these MSCs had a reduced ability to inhibit Th1 differentiation and proliferation in comparison with the effect of untreated MSCs (*p* < 0.05). Regarding the ability of MSCs to inhibit the clonal expansion of IL-17-secreting (Th17) lymphocytes, we observed that untreated MSCs significantly inhibited Th17 differentiation (*p* < 0.05, Fig. 3e, f). Furthermore, Th17 cultured with MSCs showed a decrease in Th17 proliferation, although these differences were not statistically significant (Fig. 3e, f). Similar to the pattern observed with Th1 cells, MSCs pretreated with LPS showed a decreased effect on Th17 differentiation compared with that observed in untreated MSCs and MSCs pretreated with poly(I:C) (*p* < 0.05, Fig. 3e, f). Finally, MSCs pretreated with LPS exhibited increased Th17 proliferation compared with the effect of MSCs pretreated with poly(I:C) (*p* < 0.05, Fig. 3 g, h). These results showed that brief pretreatment of murine MSCs with poly(I:C) or LPS induces different and opposing effects on Th1 and Th17 cell differentiation and proliferation, suggesting that stimulation of murine MSCs with TLRs can modulate the cells’ in-vitro immunosuppressive capacity against T-helper cell subsets.

### Brief in-vitro pretreatment of MSCs with poly(I:C) or LPS induces distinct and opposing immunomodulatory effects on EAE

**Fig. 4:**
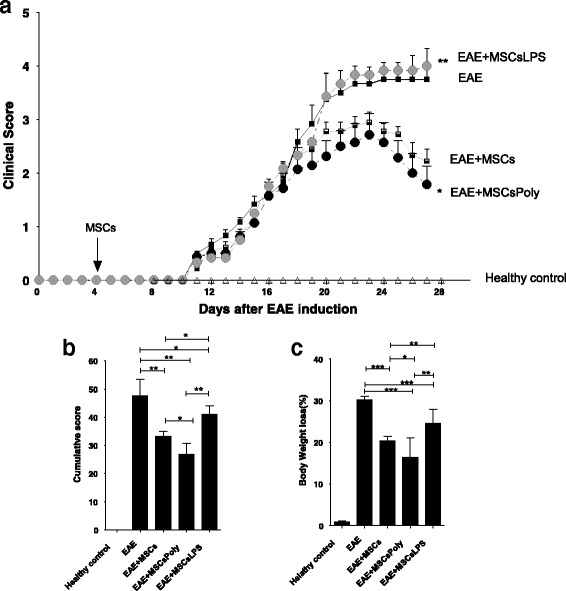
MSCs treated with poly(I:C) or LPS generate distinct immunomodulatory effects in an EAE model. Data show EAE clinical signs according to the different treatments of MSCs (either pretreated or not with TLR3 and TLR4 ligands). EAE was induced in C57BL/6 mice (*n* = 10/group) by subcutaneous immunization with 50 μg of MOG_35–55_ peptide, as described in Methods. MSC controls and MSCs pretreated with poly(I:C) or LPS were injected i.p. at day 4 (2 × 10^6^/mouse) as described in Methods. (**a**) Scores were measured daily at the same time for 27 days and given a value of 0–5 according to loss of mobility in the lower and upper extremities. (**b**) The sum of the scores from day 10 to the end of the experiment was pooled by treatment group. Poly(I:C)-pretreated MSCs show a significantly lower cumulative score than that of EAE control mice. (**c**) Body weight loss (%), measured daily and clustered by treatment. A Wilcoxon rank test was used for comparisons with the untreated MSCs. *EAE + MSCsPoly compared with EAE and **EAE + MSCsLPS compared with EAE + MSCs with the matched pairs test, *p* < 0.001. *MSCsPoly* MSCs pretreated with poly(I:C) for 1 hour, *MSCsLPS* MSCs pretreated with LPS for 1 hour

To elucidate the therapeutic effect of untreated MSCs or MSCs pretreated with poly(I:C) or LPS, we induced EAE in C57BL/6 mice using MOG_35–55_ immunization as described previously [12]. MSCs were injected i.p. (2 × 10^6^ cells/mice) 4 days after EAE induction, and the clinical scores and body weight loss were recorded daily until day 27 (Fig. 4a). Control EAE mice showed the first clinical signs at day 10 post immunization (onset), reached a peak at day 21, and then presented a stable disease course until day 27, as we observed previously [12]. Consistent with previous reports [12], the administration of untreated MSCs before the onset of clinical signs significantly decreased the clinical signs of EAE compared with control treatment (*p* < 0.05, Fig. 4a). The administration of MSCs pretreated with poly(I:C) for 1 hour generated a nonsignificant increase of the therapeutic effect on EAE clinical scores relative to untreated MSCs, decreasing the progress of the disease even further (Fig. 4a). In contrast, MSCs pretreated with LPS completely reversed the protective effect of MSCs against EAE, showing a similar trend in the clinical manifestations of the disease to that observed in the control EAE mice (Fig. 4a). These results were confirmed by analyzing the cumulative EAE score, which showed that untreated MSCs significantly decreased the clinical signs of EAE compared with the control treatment (*p* < 0.001, Fig. 4b). On the other hand, MSCs pretreated with poly(:C) were even more potent than untreated MSCs in significantly inhibiting the cumulative EAE score compared with the score of the control EAE mice (Fig. 4b). In contrast, MSCs pretreated with LPS significantly reversed the trend observed in the cumulative score induced by untreated MSCs or MSCs pretreated with poly(I:C) (*p* < 0.05) (Fig. 4b).

Similarly, analysis of body weight loss demonstrated that untreated MSCs and MSCs retreated with poly(I:C) resulted in significantly less weight loss compared with the control (*p* < 0.05, Fig. 4c). Finally, the administration of MSCs pretreated with LPS reversed the effect on body weight loss induced by untreated MSCs and MSCs pretreated with poly(I:C) (*p* < 0.05, Fig. 4c). These data consistently demonstrate that MSCs pretreated with poly(I:C) reduce the clinical signs of EAE and that pretreatment of MSCs with LPS reverses this effect, suggesting that specific TLR activation can alter the immunomodulatory capacity of MSCs in vivo.

**Fig. 5:**
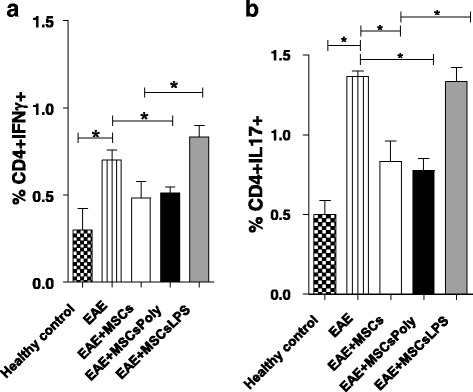
Pretreatment of MSCs with poly(I:C) and LPS generated a differential modulation of Th1 and Th17 cells in EAE mice. Lymph nodes were removed from different groups of treatments at day 27. (**a**) Th1 detection using CD4^+^IFN-γ for the five groups of mice. (**b**) Th17 detection (CD4^+^IL-17^+^) for the five groups of mice. *Bars* represent the mean ± SEM, significant differences calculated using *t* tests (*p < 0.05, **p < 0.001). *MSCsPoly* MSCs pretreated with poly(I:C) for 1 hour, *MSCsLPS* MSCs pretreated with LPS for 1 hour

We next evaluated whether the administration of untreated MSCs could affect Th1 and Th17 cell subsets in EAE mice. Percentages of Th1 and Th17 subsets were analyzed in lymph nodes samples of EAE mice by flow cytometry as described in Methods. As expected, treatment with MSCs decreased Th1 and Th17 subsets in the lymph nodes of EAE mice. We found a significant effect on the Th17 subset (*p* < 0.05) and a decrease in the Th1 subset. Interestingly, pretreatment of MSCs with poly(I:C) was able to significantly decrease both the Th1 and Th17 subsets (*p* < 0.05). In contrast, the administration of MSCs pretreated with LPS completely reversed the effect of MSCs on the Th1 and Th17 subsets (*p* < 0.05, Fig. 5a, b). We observed higher percentages of Th1 and Th17 in this group, similar to percentages found in the EAE mice without any treatment.

## Discussion

Recently years, stem cell treatments have become an important therapeutic strategy for the treatment of various proinflammatory and autoimmune diseases because of their powerful immunomodulatory properties via the suppression of T cells, B cells, natural killer (NK) cells, and antigen presenting cells [26, 27]. Such immunological effects have been shown primarily in vitro but also in vivo, in a number of experimental disease models such as EAE [10–12], CIA [8], and experimental colitis [9, 28]. Despite the in-vitro and in-vivo evidence for a therapeutic effect of MSCs, their precise mechanism of action and the profile of their adverse effects as immunomodulatory agents are still poorly understood.

Recently, it has been demonstrated that stimulation of human MSCs with poly(I:C) and LPS induces activation of NF-kB, mitogen-activated protein kinases (MAPK), and protein kinase B (AKT) signaling pathways. Activation of these pathways was associated with the induction and secretion of different patterns of cytokines and chemokines, suggesting that LPS could promote the activation of immune responses while poly(I:C) could suppress it [29]. Similarly, Waterman et al. [20] demonstrated that human MSCs polarize into a proinflammatory or anti-inflammatory phenotypes, according to the specific TLR3 or TLR4 activation in vitro. This functional phenotype was also shown in vivo, in experimental models of diabetes [30] and ovarian cancer [31]. These findings suggest that pretreatment of MSCs with TLRs could be a powerful and innovative therapeutic tool for the treatment of autoimmune and proinflammatory pathologies. In the present study, we evaluated the immunomodulatory effect of murine MSCs after treatment with TLR3 and TLR4 agonists in vitro and in a mouse model of multiple sclerosis. Our results demonstrated that pretreatment of MSCs with poly(I:C) enhances their immunosuppressive capacity in vitro and that intraperitoneal injection of these MSCs significantly reduces the severity of EAE. In contrast, LPS pretreatment of MSCs induces a significant decrease in their immunomodulatory function in vitro and completely reverses the therapeutic immunosuppressive effect of MSCs in vivo.

Diverse studies have shown that murine MSCs express different functional TLRs, such as TLR1–TLR8 [32]. Our data showed that murine MSCs cultured to 80–90 % confluence in complete culture medium express significant levels of mRNA for TLR3 and TLR4, and that the expression level of TLR4 was higher than that of TLR3, similar to the pattern described by Pevsner-Fischer et al. [32]. In addition, we demonstrated that pretreatment of these TLRs for 1 hour with their respective agonists differentially affects the in-vitro immunosuppressive capacity of murine MSCs. First, we observed that untreated MSCs were functionally capable of inhibiting the proliferation of activated T cells, confirming what has been published previously [7]. Once the inhibitory capacity of the MSCs on T-cell proliferation was confirmed, we evaluated the effect of MSCs pretreated for 1 hour with poly(I:C) or LPS. MSCs pretreated with poly(I:C) were able to significantly increase the inhibitory capacity of MSCs on T-cell proliferation by approximately 33 % with respect to untreated MSCs. Conversely, MSCs pretreated with LPS completely reversed the immunosuppressive effect of untreated MSCs and induced a significant, and dose-dependent, increase in T-cell proliferation.

These results demonstrate that brief, in-vitro LPS stimulation of murine MSCs induces a proinflammatory phenotype, similar to the effects previously shown by Waterman et al. [20], using human MSCs.

To better understand the effect of activation of TLR ligands on the immunomodulatory activity of MSCs, we measured NO production in the absence or presence of splenocytes stimulated with ConA as well as the expression and levels of proinflammatory cytokine IL-6. In the absence of splenocytes, no differences were observed in NO secreted by untreated or pretreated MSCs. However, in the presence of splenocytes, we detected a significant increase in NO production induced by MSCs pretreated with poly(I:C) but not by those pretreated with LPS, which had lower NO production compared with untreated MSCs. On the other hand, our results indicated that the expression of IL-6 increased after stimulation of MSCs with LPS and was inhibited after stimulation of MSCs with poly(I:C). Taken together, these data provide evidence of a based anti-inflammatory phenotype for MSCs pretreated with poly(I:C) and an opposite, proinflammatory phenotype for MSCs stimulated with LPS, which show a loss of capacity to inhibit T-cell proliferation, a higher expression of IL-6, and nonsignificant NO secretion. MSCs have been identified as immunomodulating cells because they inhibit the generation and function of Th1 and Th17 cells and increase Treg cell formation [33–36]. Previous studies from our laboratory showed that MSCs cocultured with CD4^+^ T cells grown in conditions polarizing them towards Th1 or Th17 lineages exert strong Th1 immunosuppression but have little effect on Th17 cells [22, 25]. Here, we evaluated the immunomodulatory effect of TLR3 and TLR4-pretreated MSCs on Th1 and Th17 differentiation and proliferation in vitro. We observed a strong capacity of MSCs pretreated with poly(I:C) to inhibit Th1 and Th17 differentiation and proliferation, which was even more pronounced than the effect of untreated MSCs. Conversely, MSCs pretreated with LPS showed a diminished capacity to inhibit Th1 and Th17 differentiation and proliferation.

Recently, we studied the therapeutic effect of MSC administration on EAE, showing that the injection of MSCs at the time of disease onset induces a significant improvement in the clinical signs of the disease [12]. In the present study, using the same mouse model, we studied whether the administration of MSCs pretreated with poly(I:C) or LPS generated distinct therapeutic effects in vivo. Our results demonstrated that MSCs pretreated with poly(I:C) significantly reduce the clinical signs of EAE and that pretreatment of MSCs with LPS completely reverses the therapeutic immunosuppressive effect of MSCs. Furthermore, when we evaluated the cumulative score and the weight loss of the animals in each group, we found the same pattern that again highlighted the ability of MSCs stimulated with poly(I:C) to increase the immunosuppressive capacity of the MSCs. Poly(I:C) stimulation generated a decrease in the score and weight loss in the treated animals, while LPS caused an increase in clinical signs and a high percentage of weight loss in animals. In addition, we investigated the relationship between the treatments of the animals with respect to Th1 and Th17 proinflammatory cell subsets in the lymph node of EAE mice as a way to account for the observed results. We found a significant decrease of the Th1 and Th17 subsets induced by the administration of untreated MSCs, although these differences were significant only in the case of Th17 cells. No significant differences were observed in the expression of Th1 and Th17 cells when EAE mice were injected with poly(I:C)-pretreated MSCs in comparison with the expression in untreated MSCs. In contrast, the treatment of EAE mice with LPS-pretreated MSCs completely reversed the effect on the Th1 and Th17 subset cells induced by untreated MSCs.

## Conclusions

In summary, for the first time we found that murine MSCs polarize into two distinct phenotypes following in-vitro specific TLR activation, as observed in humans. TLR3 stimulation specifically leads to enhancement of the immunosuppressive capacity to inhibit the proliferation of splenocytes and the differentiation and proliferation of Th1 and Th17 in vitro. Meanwhile, TLR4 stimulation completely reverses these immunomodulatory effects. Secondly, we also examined these phenotypes in the context of the autoimmune disease model of multiple sclerosis, where pretreatment of murine MSCs with TLR3 and TLR4 agonists generates distinct and opposing immunomodulatory effects on EAE. Our findings are important to better define strategies of cell-based therapies for proinflammatory and autoimmune diseases.

## Abbreviations

BM-MSC: Bone marrow-derived mesenchymal stem cell
EAE: Experimental autoimmune encephalomyelitis
IDO: Indoleamine 2,3-dioxygenase
IFN-γ: Interferon gamma
IL: Interleukin
LPS: Lipopolysaccharide
MLR: Mixed lymphocyte reaction
MSC: Mesenchymal stem cell
NO: Nitric oxide
Poly(I:C): Polyinosinic:polycytidylic acid
RT-qPCR: Quantitative real-time polymerase chain reaction
TGF-β: Transforming growth factor beta
Th1: T helper 1
Th17: T helper 17
TLR: Toll-like receptor
TNF-α: tumor necrosis factor alpha
Treg: T regulatory cell
αMEM: alpha minimal essential medium
